# Left ventricular systolic dysfunction predicted by artificial intelligence using the electrocardiogram in Chagas disease patients–The SaMi-Trop cohort

**DOI:** 10.1371/journal.pntd.0009974

**Published:** 2021-12-06

**Authors:** Bruno Oliveira de Figueiredo Brito, Zachi I. Attia, Larissa Natany A. Martins, Pablo Perel, Maria Carmo P. Nunes, Ester Cerdeira Sabino, Clareci Silva Cardoso, Ariela Mota Ferreira, Paulo R. Gomes, Antonio Luiz Pinho Ribeiro, Francisco Lopez-Jimenez

**Affiliations:** 1 Faculdade de Medicina, Universidade Federal de Minas Gerais, Belo Horizonte, Brazil; 2 Department of Cardiovascular Medicine, Mayo Clinic, Rochester, Minnesota, United States of America; 3 Telehealth Center, Hospital das Clínicas, Universidade Federal de Minas Gerais, Belo Horizonte, Brazil; 4 Department of Statistics, Instituto de Ciência Exatas, Universidade Federal de Minas Gerais, Belo Horizonte, Brazil; 5 London School of Hygiene and Tropical Medicine, London, United Kingdom; 6 Instituto de Medicina Tropical da Faculdade de Medicina da Universidade de São Paulo, São Paulo, Brazil; 7 Federal University of São João del-Rei, Divinópolis, Brazil; 8 Graduate Program in Health Sciences, State University of Montes Claros, Montes Claros, Minas Gerais, Brazil; Baylor College of Medicine, UNITED STATES

## Abstract

**Background:**

Left ventricular systolic dysfunction (LVSD) in Chagas disease (ChD) is relatively common and its treatment using low-cost drugs can improve symptoms and reduce mortality. Recently, an artificial intelligence (AI)-enabled ECG algorithm showed excellent accuracy to detect LVSD in a general population, but its accuracy in ChD has not been tested.

**Objective:**

To analyze the ability of AI to recognize LVSD in patients with ChD, defined as a left ventricular ejection fraction determined by the Echocardiogram ≤ 40%.

**Methodology/principal findings:**

This is a cross-sectional study of ECG obtained from a large cohort of patients with ChD named São Paulo-Minas Gerais Tropical Medicine Research Center (SaMi-Trop) Study. The digital ECGs of the participants were submitted to the analysis of the trained machine to detect LVSD. The diagnostic performance of the AI-enabled ECG to detect LVSD was tested using an echocardiogram as the gold standard to detect LVSD, defined as an ejection fraction <40%. The model was enriched with NT-proBNP plasma levels, male sex, and QRS ≥ 120ms.

Among the 1,304 participants of this study, 67% were women, median age of 60; there were 93 (7.1%) individuals with LVSD. Most patients had major ECG abnormalities (59.5%). The AI algorithm identified LVSD among ChD patients with an odds ratio of 63.3 (95% CI 32.3–128.9), a sensitivity of 73%, a specificity of 83%, an overall accuracy of 83%, and a negative predictive value of 97%; the AUC was 0.839. The model adjusted for the male sex and QRS ≥ 120ms improved the AUC to 0.859. The model adjusted for the male sex and elevated NT-proBNP had a higher accuracy of 0.89 and an AUC of 0.874.

**Conclusion:**

The AI analysis of the ECG of Chagas disease patients can be transformed into a powerful tool for the recognition of LVSD.

## Author summary

Chagas disease (ChD) is caused by the protozoan parasite *Trypanosoma cruzi* and continues to be a health problem despite the control of its transmission. ChD is a heterogeneous condition with a wide variation in its clinical course and prognosis. The majority (60%–70%) of infected individuals remain asymptomatic throughout life. Although some develop only conduction defects and mild segmental wall motion abnormalities, others develop severe symptoms of heart failure (HF), thromboembolic phenomena, and life threatening ventricular arrhythmias. HF is one of major causes of the death of patients with ChD. There is some evidence on effective drugs against the parasite in the chronic form of the disease capable of preventing long-term adverse outcomes, but it is still limited. However low-cost medications are able to reduce mortality and improve the quality of life of patients with HF. Because of the lack of tertiary care facilities outside urban centers, an automatic diagnostic tool based on the ECG, which is a relatively simple exam without requiring human interpretation, would improve the capacity to recognize HF. Recently, digital signals of the electrocardiogram were recognized by Artificial Intelligence (AI) and associated with an excellent accuracy for HF in the general population. Our results demonstrate that AI-ECG could ensure a rapid recognition of HF in patients who require a referral to a cardiologist and the use of disease-modifying drugs. AI can be used as a powerful public heath tool, it can transform the lives of 6 million patients with ChD worldwide, and it may well have a formidable impact on patient management and prognosis.

## Introduction

Chagas disease (ChD) is caused by the protozoan parasite *Trypanosoma cruzi* and continues to be a health problem despite the control of its transmission. The World Health Organization (WHO) estimates that approximately six million people have been infected, particularly in Latin America, where the disease is endemic [1,2]. Left ventricular systolic dysfunction (LVSD) is the most important predictor of mortality in Chagas Cardiomyopathy (ChCM). The treatment of ChCM is based on relatively low-cost drugs, which can improve symptoms and survival [2]. Unfortunately, the diagnosis of LVSD requires advanced diagnostic testing, such as echocardiography (echo), computer tomography, or magnetic resonance, none of which is readily accessible to most patients with ChD in most endemic areas.

The electrocardiogram (ECG) has played an important role in the detection and evaluation of ChCM since the early years of its description [3]. Moreover, the ECG is a low-cost, widely available exam. Many of the ECG abnormalities have been described as associated with a worse prognosis in ChCM and the need of further clinical evaluation and care [4–8]. However, only a fraction of those with abnormal ECG presented LVSD, which can also occur in subjects with minor ECG abnormalities, limiting its role in the recognition of patients that are candidates for the use of drugs that can improve the prognosis in LVSD, such as betablockers and ACE inhibitors [8].

Machine learning is a field of Artificial Intelligence (AI) based on computational algorithms that allow computers to learn directly from data, without being explicitly programmed [9]. AI properly trained neural networks can recognize digital patterns that are not yet available to human knowledge. Recently, digital signals of the ECG were recognized by AI and associated with an excellent accuracy to detect LVSD in the general population [10].

The present study analyzes the ability of an AI-ECG algorithm [10] to identify LVSD, defined as a left ventricular ejection fraction (LVEF) of less than 40% in patients with ChD [11,12]. LVEF was obtained by means of an echocardiogram and calculated according to the Simpsons method. A previously developed AI-ECG algorithm to detect LVSD in the general population [10] was applied to the patients with ChD from the São Paulo-Minas Gerais Tropical Medicine Research Center cohort (SaMi-Trop Study). The AI-predicted LVSD performance was then tested in different clinical models.

## Material and methods

### Ethics statement

This research was approved by the Brazilian National Institutional Review Board (CONEP), logged under protocol number 179.685/2012. All participants provided written informed consent.

### Study design

This is a cross-sectional study of ECG obtained from the second wave of a large cohort of patients with Chagas disease from an endemic area, called the SaMi-Trop Study, which is described elsewhere [13]. The SaMi-Trop consists of a network of collaborating scientists in the Brazilian States of Minas Gerais and São Paulo and has been established to develop and conduct research projects on ChD. The SaMi-Trop study is a prospective cohort begun in 2013 which selected patients with ChD in 21 municipalities in the northern part of the state of Minas Gerais, where the prevalence of patients with chronic ChCM was expected to be high [13]. The cohort was established by using patients under the care of the Telehealth Network of Minas Gerais, a program designed to support primary care in Minas Gerais, Brazil. Eligible patients were selected based on the ECG results performed in 2011–2012 by the Telehealth Network, henceforth called index ECG. Only patients who fulfilled all of the following inclusion criteria were selected: (1) self-reported Chagas disease; (2) presence of any abnormalities in the index ECG, and (3) aged 19 years or over. The exclusion criteria included pregnancy or breast feeding, and any life-threatening disease with an ominous prognosis that suggested a life expectancy of <2 years. From the total of 4,689 patients eligible for the study, 2,157 were located and had the baseline assessment completed.

Subsequently, all participants were tested for *T*. *cruzi* infection using 2 serologic assays, a *T*. *cruzi* lysate-based enzyme immunoassay and a recombinant enzyme immunoassay (Wiener Lab). The final cohort included only patients with two positive serologic tests against *T*. *cruzi*, resulting in 1,959 individuals in 2013–2014.

**Fig 1:**
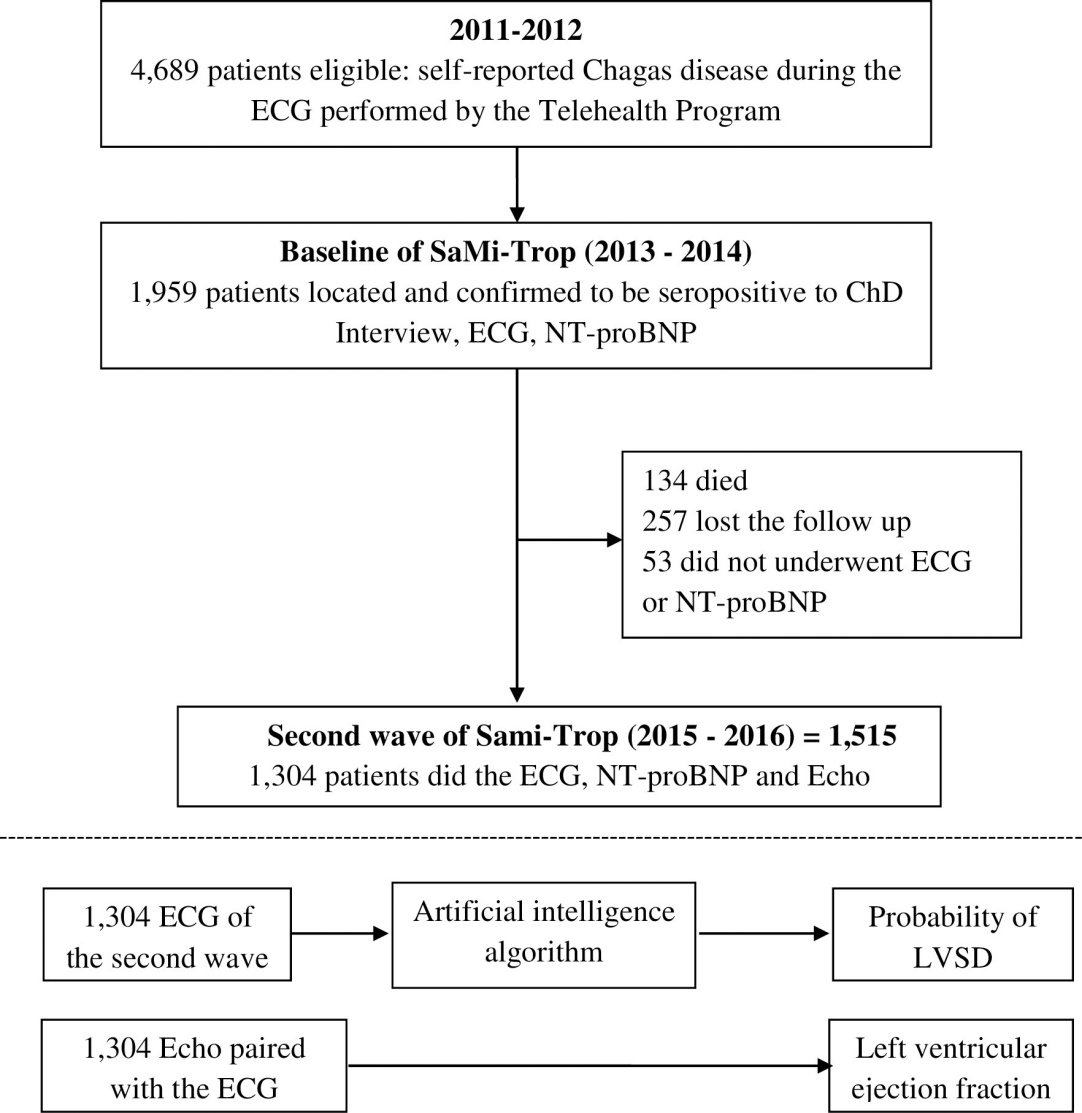
Flow chart describing study design.

The baseline visit was performed between 2013 and 2014 at public health primary care units by previously trained staff. The patients were interviewed using a standardized questionnaire, had a blood sample collected and an ECG evaluation. Although the sample of this cohort was selected among eligible patients with an ECG performed in 2011–2012 by the Telehealth Network of Minas Gerais Brazil, all ECGs were repeated for the admission in the cohort, performed by previously trained staff to guarantee its uniformity. After two years of follow-up, all patients were contacted for a second visit at public health primary care units (2015–2016). The evaluation included a new ECG, a health-related questionnaire, and a new NT-proBNP (N terminal pro-B-type natriuretic peptide, Roche Diagnostics) testing. The NT-proBNP test was obtained and categorized according to age-specific cut-off points for heart failure [14]. This study included patients with confirmed ChD who attended the second visit and who had the original digital tracings of the ECG. The second wave data was used because the patients underwent the Echo evaluation, the gold standard to detect LVSD, only at this moment. Fig 1 describes the design of this study.

Transthoracic echocardiography was performed using a commercially available ultrasound system (Vivid Q, GE) with standard protocols according to the recommendations set forth by the American Society of Echocardiography. The exam was generally performed the same day of the ECG and acquired images were stored digitally and transferred to be analyzed offline with EchoPac software (GE Medical). Left ventricular ejection fraction was calculated according to the Simpsons method [15].

### The electrocardiogram and artificial intelligence evaluation

A resting 12-lead ECG was recorded using an ECG PC machine (TEB, São Paulo, Brazil). The ECG recordings were sent electronically to the Telehealth system. The ECG’s measures were automatically analyzed using the University of Glasgow’s ECG analysis program (release 28.5, issued on January 2014), and a clinical report was sent back to the primary healthcare unit. For research goals, all ECGs were manually reviewed and codified by a trained cardiologist using the Minnesota Code (MC) criteria [16].

The ECG of these ChD individuals were submitted to the analysis of the previously trained machine to recognize LVSD in the general population, as previously described. As the original model expects two seconds, 12-lead ECGs sampled at 500Hz, represented a matrix of 1000 x 12 samples for each ECG. All ECGs from this study were first resampled to 500Hz, and then filtered using a Butterworth bandpass filter of 0.5-100Hz. Each ECG was segmented to two second windows, with a one second overlap (0–2 seconds, 1–3 seconds, 2–4 seconds, etc.) and the average score of the AI-LVSD algorithm was used as the ECG probability of having low EF (0 means normal EF and 1 means low EF) [10].

### Statistical analysis

A descriptive analysis of the data with frequencies, means, medians of electrocardiographic variables, major ECG abnormalities, as well as other clinical variables were obtained. The Kolmogorov-Smirnov test was performed to evaluate normality. The variables with normal distribution were described as means and standard deviation, while those with asymmetric distribution were described as medians and the first to third interquartile range.

The output of the AI algorithm was the probability of LVSD of each person after the AI ECG analysis. Diagnostic performance included accuracy, sensitivity, specificity, positive predictive value (PPV), negative predictive value (NPV), and the area under the curve (AUC). The accuracy of a test is its ability to differentiate the patient and healthy cases correctly. To estimate the accuracy of a test, we should calculate the proportion of true positive and true negative in all evaluated cases. Logistic regression was applied to test the association of AI-predicted LVSD with LVSD. Different cut-off points were obtained for each model in order to maximize their sensibility or specificity. The exposure variable was AI-predicted LVSD given by a continuous probability. The covariates were elevated NT-proBNP, male sex, major ECG abnormalities, QRS ≥ 120ms, age, heart rate, and heart rate > 80 bpm. These covariates have a major importance in ChCM prognosis [8,17].

Five logistic regression models were developed to evaluate AI—predicted LVSD performance. First, a univariate model was created to identify the importance of other clinical and electrocardiographic variables that could improve AI-predicted LVSD results when applied in a real-world scenario. The present study’s models have predicted the LVSD when its outputs—a number between 0 and 1—are above a threshold. Those thresholds were chosen to determine their best sensitivity and specificity. The best calculated threshold for the AI-predicted LVSD univariate model was 0.40. In the second model, the first one was adjusted to the male sex and QRS duration ≥ 120 ms, and the best threshold was 0.15. In the third model, the first one was adjusted to the male sex and abnormal NT-proBNP, and the best threshold was 0.11. The fourth was adjusted to the male sex, QRS duration ≥ 120 ms, and abnormal NT-proBNP. The fifth model included only the patients with major electrocardiographic abnormalities, and it was adjusted to the male sex and abnormal NT-proBNP. The best threshold for the fifth model was 0.11.

A further analysis of the third model was conducted to compare the clinical and electrocardiographic characteristics of the false positive individuals with the true positive and of the false negative with the true negative. The Mann-Whitney test was applied to compare the medians. To compare categorical variables, the Chi-squared and the Fisher tests were used.

## Results

Among the 1,304 participants of this study, the median age was 60 (51–69), of which 872 (67%) were women. There were 93 (7.1%) individuals with LVSD. NT-proBNP was high in 148 (11.3%) individuals of the study population. Most of the population presented major ECG abnormalities (59.5%). Baseline characteristics of the study participants are shown in Table 1.

**Table 1:** Clinical characteristics of the Chagas disease population by gender (n = 1,304).

Characteristics	Total (n = 1,304)	Men (n = 432)	Women (n = 872)
Age (years)	60 (51–69)	60 (52–69)	59 (50–69)
Ethnicity			
Black	229 (17.5)	72 (16.6)	157 (18.0)
White	292 (22.4)	100 (23.1)	192 (22.0)
Mixed	755 (58.0)	249 (57.6)	506 (58.0)
Indigenous	2 (0.1)	1 (0.2)	1 (0.1)
Literacy	741 (56.8)	238 (55.1)	503 (57.7)
Diabetes mellitus^#^	144 (11.0)	37 (8.5)	107 (12.2)
Chronic kidney disease^#^	133 (10.2)	49 (11.3)	84 (9.6)
Systemic hypertension^#^	840 (64.4)	273 (63.2)	567 (65.0)
History of myocardial infarction^#^	67 (5.1)	27 (6.2)	40 (4.5)
Previous use of benzonidazole^#^	82 (6.3)	27 (6.2)	55 (6.3)
Heart rate	64 (57–72)	62 (56–70)	65 (58–74)
QRS duration	110 (92–140)	120 (98–148)	104 (90–136)
Major Q wave	119 (9.1)	49 (11.3)	70 (8.0)
Major ST-T	151 (11.5)	41 (9.5)	110 (12.6)
Right bundle branch block	404 (31.0)	131 (30.3)	273 (31.3)
RBBB + LAH	9 (0.7)	2 (0.4)	7 (0.8)
Intraventricular block	37 (2.8)	19 (4.4)	18 (2.0)
Left bundle branch block	49 (3.7)	22 (5.1)	27 (3.1)
Premature ventricular contraction	33 (2.5)	12 (2.7)	21 (2.4)
Any major ECG abnormality	776 (59.5)	270 (62.5)	506 (58.0)
Pacemaker	54 (4.1)	30 (6.9)	24 (2.7)
Atrial fibrillation	64 (4.9)	30 (6.9)	34 (3.9)
High NT-proBNP^&^	148 (11.3)	69 (15.9)	79 (9.0)
NT-proBNP	146 (63–372)	162 (54–534)	140 (67–320)
LVEF	63 (57–66)	61 (50–65)	63 (59–66)

* Data are expressed by percentage except Age, NT-proBNP, QRS duration, heart rate, and LVEF, which are expressed by medians and quartiles.

# Self-reported data. RBBB + LAH: Right bundle branch block + Left Anterior Hemiblock; LVEF: Left Ventricular Ejection Fraction. & Number of individuals and the percentage of them with high NT-proBNP levels.

**Fig 2:**
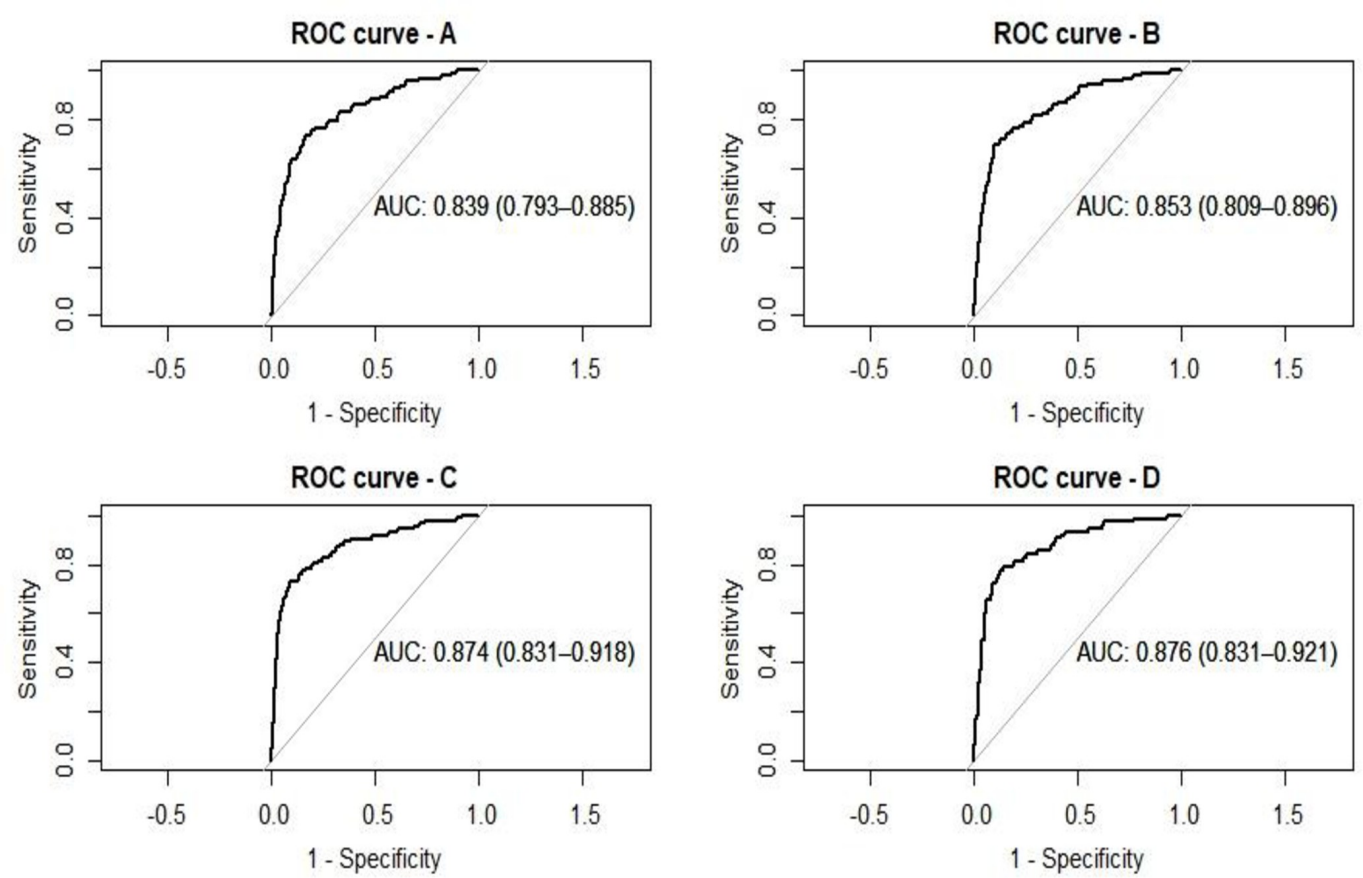
Receiver Operating Characteristic (ROC) curves for AI prediction of LVSD (n = 1,304). A: All patients (n = 1,304), univariate analysis; B: All patients (n = 1,304), adjusted for the male sex and QRS ≥ 120ms; C: All patients (n = 1,304), adjusted for the male sex and NT-proBNP; D: Only the patients with major ECG abnormalities (n = 776), adjusted for the male sex and NT-proBNP.

In the univariate model, AI has an odds ratio of 63.3 (95% CI 32.3–128.9) to predict LVSD. Using the optimal cutoff value of probability to detect LVSD (P = 0.40), the AI algorithm was able to identify LVSD among patients with ChD with a sensitivity of 0.73 (0.71–0.75), specificity of 0.83 (0.74–0.92), overall accuracy of 0.83 (0.80–0.85), negative predictive value of 0.97 (0.92–1.00), and a positive predictive value of 0.25 (0.24–0.26). The AUC was 0.839 (0.793–0.885), as observed in Fig 2. Other important clinical variables associated with LVSD were high NT-proBNP, QRS ≥ 120ms, and the male sex, in this order, as seen in Table 2.

**Table 2:** Clinical variables associated with left ventricular systolic dysfunction in univariate logistic regression (n = 1,304).

Variables	Odds Ratio	(95% CI)
Artificial Intelligence-based ECG LVSD	63.35	(32.33–128.90)
High NT-proBNP	22.42	(13.98–36.57)
Male sex	4.10	(2.65–6.44)
Major ECG abnormalities	3.26	(1.95–5.77)
QRS ≥ 120ms	4.55	(2.84–7.55)
Age	0.99	(0.98–1.01)
Heart rate	1.00	(0.98–1.02)
Heart Rate > 80bpm	1.00	(0.48–1.90)

When incorporating sex into the model and QRS duration ≥ 120ms, the AUC improved to 0.853 (0.809–0.896) and had a higher specificity of 0.90 (0.81–0.99). After incorporating sex and NT-proBNP, the accuracy increased to 0.89 (0.88–0.91), with a higher specificity of 0.91 (0.82–1.00) and an AUC of 0.874 (0.831–0.918). The QRS duration ≥ 120ms was not significant in the fourth model which also included the male sex and NT-proBNP; further analyses were not conducted. When only the AI results of the individuals with major ECG abnormalities were analyzed and adjusted for sex and NT-proBNP, there was a slight increase in the odds ratio to 9.80 (3.73–26.64) and sensitivity to 0.78 (0.76–0.81). There was no improvement in the AUC, PPV, and NPV when compared to the third model. The other properties of these proposed tests are demonstrated in Table 3. Fig 2 shows the Receiver Operating Characteristic (ROC) curves of each model.

**Table 3:** Logistic regression models to predict the ability of AI to recognize LVEF and its performance in clinical practice scenarios.

	A- AI Univariate analysis (n = 1,304)	B- Adjusted for the male sex and QRS ≥ 120ms (n = 1,304)	C- Adjusted for the male sex and NT-proBNP (n = 1,304)	D- Major ECG abnormalities adjusted for the male sex and NT-proBNP (n = 776)
Odds Ratio (95% CI)	63.35 (32.33–128.90)	36.14 (17.26–78.37)	9.41 (4.04–22.21)	9.80 (3.73–26.64)
Accuracy	0.83 (0.80–0.85)	0.89 (0.87–0.90)	0.89 (0.88–0.91)	0.85 (0.83–0.88)
Sensitivity	0.73 (0.71–0.75)	0.69 (0.68–0.71)	0.73 (0.71–0.74)	0.78 (0.76–0.81)
Specificity	0.83 (0.74–0.92)	0.90 (0.81–0.99)	0.91 (082–1.00)	0.86 (0.77–0.95)
PPV	0.25 (0.24–0.26)	0.35 (0.34–0.36)	0.38 (0.37–0.39)	0.38 (0.37–0.40)
NPV	0.97 (0.92–1.00)	0.97 (0.90–1.00)	0.97 (0.90–1.00)	0.97 (0.89–1.00)
AUC	0.84 (0.79–0.88)	0.85 (0.81–0.87)	0.87 (0.83–0.92)	0.87 (0.83–0.92)

PPV: Positive predictive value; NPV: Negative predictive value; AUC: Area under the curve. Model A: All patients (n = 1,304), univariate analysis; Model B: All patients (n = 1,304), adjusted for the male sex and QRS ≥ 120ms; Model C (n = 1,304): All patients, adjusted for the male sex and NT-proBNP; Model D: Included only the patients with major ECG abnormalities (n = 776), adjusted for the male sex and NT-proBNP.

The third model had 106 false positive (FP) individuals and 67 true positives (TP), as described in Table 4. These groups had no differences in age, sex, ethnicity, and comorbidities. The TP group has a higher proportion of individuals with a high NT-proBNP (89.5%) and a pacemaker (31.3%). On the other hand, the FP has a higher proportion of major ST-T abnormalities (10.3%) and RBBB (41.5%). These groups have a similar proportion of major ECG abnormalities: 88.0% in the TP group and 90.5% in the FP group (p = 0.78).

**Table 4:** Clinical and electrocardiographic characteristics of the false positive (FP) and true positive (TP) population (n = 173).

Characteristics	FP (n = 106)	TP (n = 67)	P
Age (years)	63.5 (52.2–71.8)	59.0 (49.5–69.0)	0.08
Male sex	69 (65.1)	45 (67.1)	0.91
Ethnicity			0.40
White	25 (23.6)	17 (25.3)	
Mixed	63 (59.4)	34 (50.7)	
Black	17 (16.0)	13 (19.4)	
High cholesterol	16 (15.1)	11 (16.4)	0.84
Diabetes	11 (10.3)	4 (5.9)	0.47
Chronic kidney disease	13 (12.2)	13 (19.4)	0.34
Systemic hypertension	72 (68.0)	45 (67.1)	0.76
Myocardial infarction	12 (11.3)	7 (10.4)	0.98
Propranolol use	4 (3.7)	1 (1.5)	0.65
Atenolol use	6 (5.6)	0 (0.0)	0.82
Amiodarone use	21 (19.8)	22 (32.8)	0.11
Previous use of benzonidazol	11 (10.3)	11 (16.4)	0.14
High NT-proBNP	70 (66.0)	60 (89.5)	0.001
Heart rate	62.0 (56.0–69.8)	64.5 (59.0–70.8)	0.46
Major Q wave	28 (26.4)	15 (22.4)	0.67
Major isolated ST—T	11 (10.3)	0 (0.0)	< 0.001
RBBB	44 (41.5)	17 (25.3)	0.04
RBBB + AFB	0 (0.0)	0 (0.0)	-
LBBB	14 (13.2)	7 (10.4)	0.76
Intraventricular block	10 (9.4)	11 (16.4)	0.26
Pacemaker	11 (10.3)	21 (31.3)	0.001
Atrial fibrillation	21 (19.8)	8 (11.9)	0.25
Atrial Flutter	2 (2.0)	2 (3.0)	0.64
PVC	8 (7.5)	4 (5.9)	0.77
LVH	1 (0.9)	0 (0.0)	1.00
Major abnormalities	96 (90.5)	59 (88.0)	0.78

VPB: Ventricular premature beats; RBBB; Right Bundle Branch Block; LBBB: Left Bundle Branch Block; LVH: Left ventricular hypertrophy

The third model also had 25 false negatives (FN) and 1,075 true negatives (TN), as described in Table 5. The FN group has a higher proportion of men and a slightly higher, but not significant, proportion of major ECG abnormalities. The FN group has a higher proportion of major Q wave abnormalities (28.0%) and major ST-T abnormalities (24.0%), although the TN group also has an important proportion of them– 9.3% and 11.1% respectively. No difference was found between the FN and TN groups when considering the clinical characteristics.

**Table 5:** Clinical and electrocardiographic characteristics of the false negative (FN) and true negative (TN) population (n = 1,098).

Characteristics	FN (n = 25)	TN (n = 1,075)	P
Age (years)	62.0 (44.0–88.0)	60.0 (19.0–95.0)	0.18
Male sex	14 (56.0)	298 (27.7)	0.004
Ethnicity			0.82
White	4 (16.0)	238 (22.1)	
Mixed	16 (64.0)	629 (58.5)	
Black	5 (20.0)	185 (17.2)	
High cholesterol	5 (20.0)	316 (29.4)	0.91
Diabetes	4 (16.0)	122 (11.3)	0.52
Chronic kidney disease	1 (4.0)	101 (9.4)	0.50
Systemic hypertension	20 (80.0)	682 (63.4)	0.16
Myocardial infarction	2 (8.0)	42 (3.9)	0,27
Atenolol use	0 (0.0)	48 (4.4)	0.62
Propranolol use	2 (8.0)	30 (2.8)	0.16
Amiodarone use	1 (4.0)	123 (11.4)	0.50
Previous use of benzonidazol	6 (24.0)	307 (28.5)	0.41
High NT-proBNP	0 (0.0)	21 (1.9)	1.00
Heart rate	68.0 (46.0–86.0)	64.0 (37.0–138.0)	0.49
Major Q wave	7 (28.0)	100 (9.3)	0.008
Major isolated ST—T	6 (24.0)	120 (11.1)	0.05
RBBB	7 (28.0)	327 (30.4)	0.96
RBBB + AFB	0 (0.0)	9 (0.83)	1.00
LBBB	2 (8.0)	25 (2.32)	0.124
Intraventricular block	0 (0.0)	25 (2.32)	0.12
Pacemaker	1 (4.0)	20 (1.8)	0.38
Atrial fibrillation	1 (4.0)	32 (2.9)	0.54
Atrial Flutter	0 (0.0)	2 (0.2)	1.00
PVC	0 (0.0)	20 (1.8)	1.00
LVH	1 (4.0)	9 (0.8)	0.20
Major abnormalities	16 (64.0)	589 (54.8)	0.48

## Discussion

This study describes the diagnostic performance of an AI-enabled ECG algorithm to detect LVSD in patients with ChD. The AI algorithm presented a high-level accuracy to recognize LVSD, together with an excellent negative predictive value, suggesting a potential role to screen (and rule out) for LVSD in this population. The incorporation of readily available information, such as sex and QRS duration, did improve the performance of the algorithm. The use of NT-proBNP level can significantly improve the AUC, the accuracy, and the specificity. The model with the addition of NT-proBNP and QRS duration ≥ 120ms in the evaluation of the same individual proved to be insignificant.

**Fig 3:**
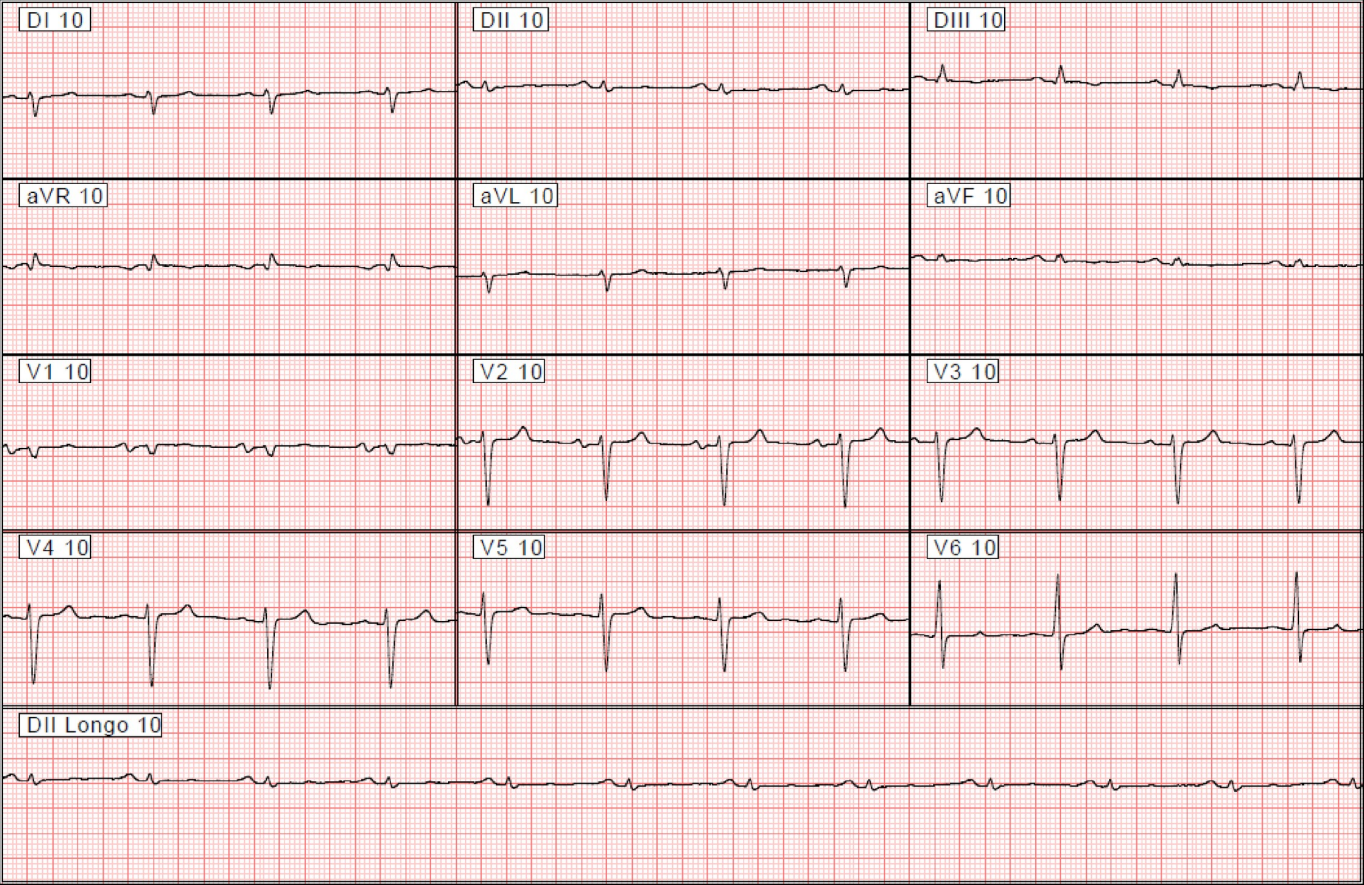
Electrocardiogram of a patient with LVSD recognized by the AI algorithm.

The ECG is the single most important test in the initial evaluation of the patients with ChD [18]. A previous study identified that the presence and the number of typical electrocardiographic abnormalities of ChD are independently associated with the severity of the ChCM [19]. That study analyzed the ECG abnormalities based on standard reading and interpretation of ECG features. An AI algorithm is expected to go beyond human skills through deep learning using artificial neural networks. This property of AI can be demonstrated by the ECG presented in the Fig 3. The ECG belongs to a patient with an ejection fraction of 28% diagnosed by the echocardiogram. However, it does not have abnormalities typical of ChCM and even a senior cardiologist would not suspect LVSD from this analysis.

Considering that the AI-ECG algorithm was originally derived and validated in the general population and not in patients with ChD, the model was enriched with basic clinical information and ECG parameters known to predict ChCM with improved performance. In contrast to the previous study [10] the male sex was associated with LVSD and improved the test specificity. A previous prognostic study of ChCM patients has already identified a worse prognosis of the male gender [20]. The accuracy, specificity, and AUC of AI-predicted LVSD were improved by the addition of QRS ≥ 120ms to the evaluation. This is in accordance with Ribeiro et al., who demonstrated that QRS duration was significantly correlated to LVSD and to left ventricular enlargement, recognizing that a small proportion of patients with narrow QRS can still have LVSD [21]. However, the best model includes NT-proBNP, a biomarker associated with the presence of LVSD in the general population and in ChD, and is strongly related to the risk of death in ChD [17]. Although NT-proBNP is considered a surrogate marker of LVSD [22,23], it is not readily available in most of clinical scenarios involving ChD.

The AI-ECG has a high NPV to rule out the presence of LVSD in patients with ChD in all the models. However, the PPV is quite low for all the models, even that which included the NT-proBNP. The positive predictive value will depend on the prevalence of LVSD in the population, in this case, 7%. The prevalence of LVSD in the SaMi-Trop cohort is representative of a community-based population with ChD [24]. The fifth model did not significantly change the PPV and the other test characteristics, although it was applied only to the results of individuals with major ECG abnormalities. There are most likely some common characteristics between the major electrocardiographic abnormalities and the alterations considered by the AI-ECG algorithm. In addition, the association between the presence of major ECG changes and LVSD is already known [19]. Assuming that the metrics performance remains, if the AI-ECG were applied to a population with a higher prevalence of LVSD, the PPV would be improved. It is also important to highlight that our study did not evaluate the clinical history, symptoms, and clinical examination of the patients, which could help to better estimate the pre-test probability and help to optimize the positive predictive value of the AI-ECG when applied in a specific clinical scenario. Although there is no difference in the clinical characteristics and the comorbidities between the FP and TP groups, the high proportion of major ECG abnormalities and high NT-proBNP in the FP group is noticeable. The major ECG abnormalities are associated with LVSD [19] and mortality [4]. High NT-proBNP levels may indicate that some of them have myocardial fibrosis, even if asymptomatic and without LVSD [25]. It can therefore be hypothesized that AI-ECG, in the FP group, might have recognized some patients that can develop LVSD during a long-term follow-up, a hypothesis that will be tested in the third visit of the same cohort.

There is a small proportion of FN individuals compared to the TN group. The FN group has a higher proportion of men, major Q wave abnormalities, and major isolated ST-T abnormalities that are related to LVSD and death in ChD [4,8]. These ECG abnormalities are also associated with myocardial infarction [26] and, in the clinical setting, those individuals should also be evaluated with an echocardiogram. It must be considered that the TN group has a high proportion of major abnormalities, indicating that the AI-ECG used digital information other than the previously established knowledge about ECG.

ChCM affects 20–40% of patients in the chronic phase of the ChD [2]. Compared to the other cardiomyopathies, individuals with ChCM have a higher mortality, a worse quality of life, and a higher number of hospitalizations [27,28]. LVSD, usually expressed by a low LVEF, which is the most important predictor of death in ChD [18]. Routine clinical practice includes a combination of beta-blockers, angiotensin-converting enzyme inhibitors, or angiotensin receptor blockers and anticoagulation, which are low-cost medications and are able to reduce mortality and improve one’s quality of life [18,29]. However, it is a great challenge to increase the access of most individuals with ChCM to this treatment.

Like other neglected tropical diseases, ChD is a chronic, stigmatizing condition, closely associated with poverty, and many patients live in remote areas of Latin America [30]. This limits the clinical evaluation to diagnose or rule out LVSD because the echo–and other cardiac imaging alternatives–is a scarce resource for this setting, and it is not readily available or not available at all [31]. Because of the lack of tertiary care facilities outside urban centers, an automatic diagnostic tool based on the ECG, which is a relatively simple exam that does not require human interpretation, would improve the capacity to recognize LVSD, which is in accordance to the World Heart Federation recommendations for the management of ChD [30]. AI-ECG could ensure a rapid recognition of LVSD, or at least an accurate screening system to identify patients who require a referral to a cardiologist and the use of disease-modifying drugs.

Another important characteristic of our AI algorithm is that it uses an inexpensive, standardized, ubiquitous test as its input—the 12-lead ECG. Although the models adjusted to NT-proBNP have the highest diagnostic accuracy than the other models, NT-proBNP is not a ubiquitous exam and, in many places, it is more expensive than the ECG and Echo, making the AI-ECG algorithm enriched with NT-proBNP less practical to be implemented in this setting. By contrast, AI analysis of the ECG is a highly desirable tool, considering that it has good diagnostic performance and is automatic and inexpensive.

Almost one century after the description of the ChCM [3], there is some evidence on effective drugs against the parasite in the chronic form of the disease capable of preventing long-term adverse outcomes, but it is still limited [18,32]. However, treating the resulting heart failure has proven to reduce mortality. The early recognition of LVSD by an AI analysis of the ECG would be one of most important advances in the evaluation and management of patients with ChD. AI can be used as a powerful public heath tool and transform the lives of six million patients with ChD around the world. Since ChD is associated with the poverty and with populations without easy medical assistance, as demonstrated by the high proportion of illiterates in this study, AI may have an impact on patient management and prognosis.

## Limitations

The AI-predicted LVSD performance with the ECG of the Chagas disease population could seem less impressive than the previously published results [10]. However, it must be considered that individuals with ChD have more complex findings on the ECG [8] than the general population that trained AI algorithms to recognize LVSD. Much fewer individuals with Chagas disease participated in this study, as compared to the 35,970 people of general population that comprised the previous training set. The logical next step to assess AI-ECG in ChD is to develop a ChD specific model using data from populations where the AI-ECG could potentially be applied. This would require a larger sample size than the current report, but could potentially have better diagnostic performance, as models perform better when applied to populations similar to those used during the training of the model. Further studies could use modelling and clinical trials to evaluate the potential impact of implementing this AI-ECG enabled algorithm to detect LVSD in patients with ChD.

The AI-ECG algorithm like any other complementary exam should be applied to shed light to a clinical questioning. This study does not contain data about the clinical history and the physical exams of the patients. This limitation of the analysis should be considered when the AI-ECG algorithm is used to test LVSD in the patients with ChD. When applied to patients with a higher pre-test probability, the algorithm may have a better positive predictive value. This hypothesis will be tested in future studies.

## Conclusion

AI analysis of the ECG of Chagas disease patients is a ubiquitous and inexpensive test and can be a powerful tool for the recognition of LVSD in the patients with ChD. Thus, public health resources can be better used, in turn resulting in improvements in the medical care provided to these patients.
