# Correction: Left ventricular systolic dysfunction predicted by artificial intelligence using the electrocardiogram in Chagas disease patients–the SaMi-Trop cohort

**DOI:** 10.1371/journal.pntd.0010488

**Published:** 2022-05-25

**Authors:** The PLOS Neglected Tropical Diseases Staff

The tenth author’s name is indexed incorrectly in PubMed. The name should be indexed as: Ribeiro ALP.
